# Optical maps of plasmids as a proxy for clonal spread of MDR bacteria: a case study of an outbreak in a rural Ethiopian hospital

**DOI:** 10.1093/jac/dkaa258

**Published:** 2020-07-12

**Authors:** Yii-Lih Lin, Tsegaye Sewunet, Sriram KK, Christian G Giske, Fredrik Westerlund

**Affiliations:** d1 Department of Biology and Biological Engineering, Chalmers University of Technology, Gothenburg, Sweden; d2 Division of Clinical Microbiology, Department of Laboratory Medicine, Karolinska Institutet, Stockholm, Sweden; d3 School of Laboratory Sciences, Department of Microbiology, Jimma University, Jimma, Ethiopia; d4 Clinical Microbiology, Karolinska University Hospital, Stockholm, Sweden

## Abstract

**Objectives:**

MDR bacteria have become a prevailing health threat worldwide. We here aimed to use optical DNA mapping (ODM) as a rapid method to trace nosocomial spread of bacterial clones and gene elements. We believe that this method has the potential to be a tool of pivotal importance for MDR control.

**Methods:**

Twenty-four *Escherichia coli* samples of ST410 from three different wards were collected at an Ethiopian hospital and their plasmids were analysed by ODM. Plasmids were specifically digested with Cas9 targeting the antibiotic resistance genes, stained by competitive binding and confined in nanochannels for imaging. The resulting intensity profiles (barcodes) for each plasmid were compared to identify potential clonal spread of resistant bacteria.

**Results:**

ODM demonstrated that a large fraction of the patients carried bacteria with a plasmid of the same origin, carrying the ESBL gene *bla*_CTX-M-15_, suggesting clonal spread. The results correlate perfectly with core genome (cg)MLST data, where bacteria with the same plasmid also had very similar cgMLST profiles.

**Conclusions:**

ODM is a rapid discriminatory method for identifying plasmids and antibiotic resistance genes. Long-range deletions/insertions, which are challenging for short-read next-generation sequencing, can be easily identified and used to trace bacterial clonal spread. We propose that plasmid typing can be a useful tool to identify clonal spread of MDR bacteria. Furthermore, the simplicity of the method enables possible future application in low- and middle-income countries.

## Introduction

The prevalence of MDR pathogens is increasing rapidly worldwide. The annual number of deaths caused by MDR infections reached ∼700000 in 2015 and could increase to an estimated 10 million in 2050, according to recent estimations.^1^ MDR infection is a severe issue in hospital settings, especially in ICUs, where many patients receive broad-spectrum antibiotics. It has thus been demonstrated that ICUs serve as long-term selection environments of MDR bacteria through selection of resistant strains in the microbiota, which can subsequently spread among patients and cause hospital-acquired infections.^2^^,^^3^ The incidence of hospital-acquired infections is 5–10 times higher in ICUs than in general wards.^4^

In Ethiopia and other East African countries, some phenotypic and a few molecular-based studies have been conducted to address the burden and consequences of antimicrobial resistance (AMR). Studies from Kenya,^5^ Tanzania,^6^ Uganda^7^ and other countries reported high prevalence of AMR to different classes of antimicrobials.^8^ A meta-analysis study on articles published from 2007 to 2017 reported that an average of 45% (range 27.5%–62.5%) of *Escherichia coli* strains were resistant to commonly prescribed antimicrobials in Ethiopia.^9^ Another study from Jimma University Medical Center, Ethiopia, in 2014 reported that 28% of *E. coli* and 70% of *Klebsiella pneumoniae* were ESBL producing.^10^ However, specific factors related to the cause and dissemination of AMR remain to be studied. Furthermore, an in-depth study requires an alternative to expensive and accurate diagnostic tools to study both local and regional outbreaks.

Current methods to characterize bacteria and their antibiotic resistance genes are either time-consuming or not able to associate the resistance genes with specific strains or genetic contexts. For example, multilocus variable-number tandem repeat analysis (MLVA) is a PCR-based assay that uses size variations of each locus to identify gene relatedness between patient isolates, but it is not discriminatory enough when faced with certain bacterial clones.^11^ Bacterial culture methods that provide phenotypic information typically require 1–3 days, and PFGE calls for lengthy sample preparation and extended gel running time, which overall requires several working days. Next-generation sequencing (NGS) provides single-base resolution, but the overall procedure from sample preparation to sequencing and bioinformatics analysis remains technically challenging and time-consuming. Novel approaches, such as long-read sequencing, are still expensive and thus less feasible, in particular in low-income settings.^12^

AMR is, to a large extent, caused by transmission of genes on mobile genetic elements, such as plasmids. Plasmids can be readily transferred among bacteria via conjugation.^13^ Typical plasmids providing resistance to multiple antibiotics are relatively large (∼50–300 kb), and the transfer of a single such plasmid can be sufficient to convert a susceptible strain to an MDR phenotype.^14^^,^^15^ When a strain has acquired a plasmid, transmission of the resistant strain between patients can occur, and detection of that plasmid may serve as a proxy for strain transmission. Techniques to classify plasmids have been used for many years, including replicon typing^16^^,^^17^ and classification of relaxases,^18^ but these often have too low resolution, for example to confirm that plasmids from two different samples are identical. Furthermore, these techniques cannot link a specific resistance gene to a certain plasmid in samples with more than one plasmid, which is common for MDR bacteria.^19^ Thus, a rapid method to identify and characterize plasmids carrying drug-resistance genes in clinical samples with high resolution can be useful to detect both clonal and horizontal dissemination of AMR genes.^20^

Optical DNA mapping (ODM) techniques directly visualize long DNA molecules at the single DNA molecule level and provide long-range information on the DNA sequence.^21^ We have reported a competitive binding based ODM method to label DNA molecules with two small molecules, YOYO-1 and netropsin, that create fluorescence intensity profiles along DNA molecules, where AT-rich regions are dark and GC-rich regions are bright.^22^^,^^23^ The labelled DNA molecules are stretched uniformly in nanofluidic channels, and the obtained fluorescence profiles are herein named ‘barcodes’. We have demonstrated that the barcodes can be used to identify plasmids from a database of all sequenced plasmids^24^ and to trace plasmids during resistance outbreaks in hospitals.^20^ We have also implemented a CRISPR/Cas9-based protocol to identify specific genes and determine on which plasmid a specific gene is located,^25^ which increases the applicability of the method.^26^^,^^27^

In this study, we analysed clinical samples from patients in three separate wards in an Ethiopian hospital. NGS identified 24 *E. coli* samples belonging to ST410 from three wards that all carried the *bla*_CTX-M-15_ resistance gene. Using ODM complemented with CRISPR/Cas9 digestion we demonstrated that up to 18 samples contained the same plasmid, either identical or with insertions or deletions. The samples with the same plasmid also had a very similar NGS profile, suggesting that plasmid mapping can be used to detect clonal bacterial spread. Samples with a less similar NGS profile did not contain the common plasmid. We discuss how ODM-based plasmid typing could be used to predict clonal spread of bacteria and therefore potentially be a useful method in low- and middle-income countries (LMICs).

## Materials and methods

### Bacterial isolates and patients

The strains were collected as part of an epidemiological study conducted in Jimma University Medical Center, Jimma, Ethiopia. The strains were isolated from clinical specimens collected from patients admitted to three different wards in the hospital [paediatric (Ped), medical (Med) and surgical (Sur)]. Thirteen strains were isolated from diarrhoeic stool collected from paediatric patients between the ages of 2 months and 10 years. Four strains were isolated from urine samples (two from the medical ward and two from the surgical ward). One strain was isolated from an infected wound in the surgical ward.

### WGS

Illumina NGS was performed for all strains investigated. A few colonies after overnight growth were lysed with a solution of BSA (0.5 mg/mL) in PBS buffer (pH 7.4). The lysate was then transferred to a 96-well plate, and the DNA was extracted with a MagNA Pure 96 (automated DNA extraction system). The extracted and purified DNA was sent for NGS to the SciLifeLab (Stockholm, Sweden). Sequencing was carried out on a short-read sequencing (Hiseq2500, Next Generation Sequencing) platform. The Nextera Library Prep Kit (MWxNXTR00x-PCR-based amplification) was used. Reads obtained were assembled on SPAdes Version 3.9, a web tool for genome assembly at CGE (https://cge.cbs.dtu.dk/services/SPAdes/) and assembled genomes were analysed for resistome and MLST using ResFinder, VirulenceFinder and MLST typing tools, respectively.

Additionally, three selected strains, Ped1, Sur3 and Med1, were subjected to MinION nanopore (Oxford, Nanopore, Oxford, UK) long-read sequencing. DNA was extracted using a Qiagen EZ1 microbial kit (according to the manufacturer’s instructions). Library preparation was done by using a ligation kit (SQK-LSK109) and with the Nanopore Native Barcoding Expansion kit (EXP-NBD-104). The sequencing was done on the MinION with a run setting of local base-call for 10 h, generating 271323 reads. The assemblies of the nanopore reads and short reads from NGS were combined through Unicycler v0.4.7 (bacterial genome assembly pipeline).

Draft genome sequences were deposited at NCBI with BioProject ID PRJNA593604.

### Plasmid preparation

The plasmid extraction procedure has been described in detail elsewhere.^25^ Briefly, the bacterial strains were incubated with selective medium (LB broth with 30 mg/L ampicillin) overnight, followed by DNA extraction using NucleoBond Xtra Midi Kit (Macherey-Nagel). The eluted DNA was then precipitated with isopropanol, washed with 70% ethanol, and reconstituted with Tris-EDTA buffer. The DNA concentration was measured using a Qubit™ dsDNA BR Assay Kit (Thermo Fisher, MA, USA).

### Sample preparation for ODM

To identify the presence of the antibiotic resistance gene *bla*_CTX-M-15_, Cas9 (PNAbio) was used to digest the circular plasmids.^25^ Cas9 forms a complex with tracrRNA and crRNA. The crRNA was designed to target *bla*_CTX-M_ group 1, where *bla*_CTX-M-15_ belongs. The Cas9 reaction was carried out with NEB buffer 3 at 37°C for 1 h. The sequence of the crRNA targeting the *bla*_CTX-M_ group 1 gene was CCGTCGCGATGTATTAGCGT.

In order to obtain the sequence-specific patterns, DNA was stained with YOYO-1 (Invitrogen) and netropsin (Sigma–Aldrich) with a typical molar ratio of 1:1.8 for YOYO-1 to DNA base pairs, and 1:70 for YOYO-1 to netropsin.^22^^,^^23^ λ-DNA (48502 bp, New England Biolabs) was added as an internal standard for size determination. The staining was done in 0.5× TBE buffer, and the samples were incubated at 50°C for 30 min.^28^ The samples were then diluted with MQ water to reduce the ionic concentration to 0.05× TBE for optimal stretching in the nanofluidic channels. To suppress photonicking, β-mercaptoethanol (Sigma–Aldrich) was added at 3% (v/v).

### Experimental procedure (microscopy)

Nanofluidic chips were fabricated using standard photo- and e-beam lithography methods and sealed by fusion bonding.^29^ The nanochannels had dimensions of 100 × 150 nm^2^ and a length of 500 μm and were aligned and connected through two microchannels, which in turn were connected to the four sample loading wells. Micro- and nanofluidic channels were pre-wetted using 0.05× TBE buffer with 3% v/v BME. Ten microlitres of sample solution was then loaded and pressed into the micro/nanochannels using nitrogen gas. The fluorescently stained plasmid DNA molecules were forced into the nanochannels and stretched for imaging. For samples with lower plasmid concentration, a pre-concentration step was applied, which began with a reduced pressure (400 mBar) to aggregate DNA molecules at the entrance of the nanochannels, and then a high-pressure pulse (2000 mBar) to push all the DNA molecules simultaneously into the nanochannels. The confined and stretched plasmid DNA molecules were then imaged using an inverted fluorescence microscope (Zeiss AxioObserver.Z1) with a 100× oil immersion objective (Zeiss, NA = 1.46) and an EMCCD camera (Photometrics Evolve). For each DNA molecule, 50 frames with an exposure time of 100 ms were recorded.

### Data analysis

Detailed analytical workflows have been described elsewhere.^25^^,^^27^ To obtain the representative barcodes for each sample, the 50 frames were first aligned and merged to generate consensus intensity profiles (barcodes). Consensus cuts by Cas9 were revealed when the individual barcodes in the consensus barcode started and ended at the same location. For each isolate, at least six barcodes were averaged to generate representative consensus barcodes, and the plasmid sizes were determined by comparing with the size of λ-DNA.

The representative consensus barcodes for each sample were then compared pairwise to investigate whether they matched entirely, or partially, the latter indicating an insertion or deletion.^20^ To identify a complete match, barcodes were stretched to the same length and compared, and if the *P* value for this comparison (*P*_comp_) was <0.01, the barcodes, and hence the plasmids, were considered the same. Complete matches were only considered when the measured size difference was less than 10%. In a second step, two barcodes were compared at their measured sizes by allowing the shorter barcode to shift along the longer one, and if the *P* value for this comparison (*P*_part_) was <0.01, then it was considered a partial match. If the comparison failed the two criteria, the barcodes, and hence plasmids, were considered not the same.

## Results

**Figure 1.**
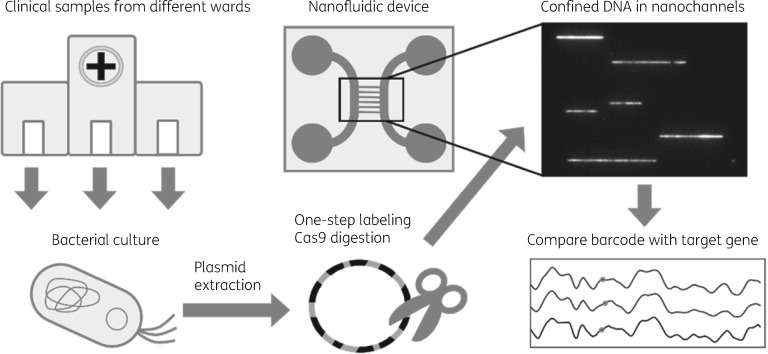
Schematic overview of ODM for plasmid characterization. Clinical samples were collected from three different wards in an Ethiopian hospital. Bacteria were cultured and the plasmids extracted. The plasmids were treated with CRISPR/Cas9 to create site-specific breaks at the gene of interest. This creates uniform, linearized DNA molecules that were then stained with YOYO-1 and netropsin to generate sequence-correlated fluorescence intensity profiles along the DNA, termed DNA barcodes. The barcodes are unique for every plasmid and can be used as a fingerprint to investigate transmission of plasmids by comparing barcodes of plasmids from different patients.

A large investigation of Gram-negative bacilli resistant to common antibiotics was performed at Jimma University Medical Center in Jimma, Ethiopia, in 2016. In the current study, we selected, based on NGS, a group of *E. coli* isolates of ST410 carrying the *bla*_CTX-M-15_ gene, originating from 24 different patients. By comparing the plasmid barcodes between patients, we wanted to identify a potentially ongoing outbreak, as then identical plasmids would be expected (Figure 1). Interestingly, the strains originated from patients that were treated in three different wards [paediatric (16), surgical (5) and medical (3)], and a potential outbreak would indicate transmission of bacteria between wards in the hospital.

**Figure 2.**
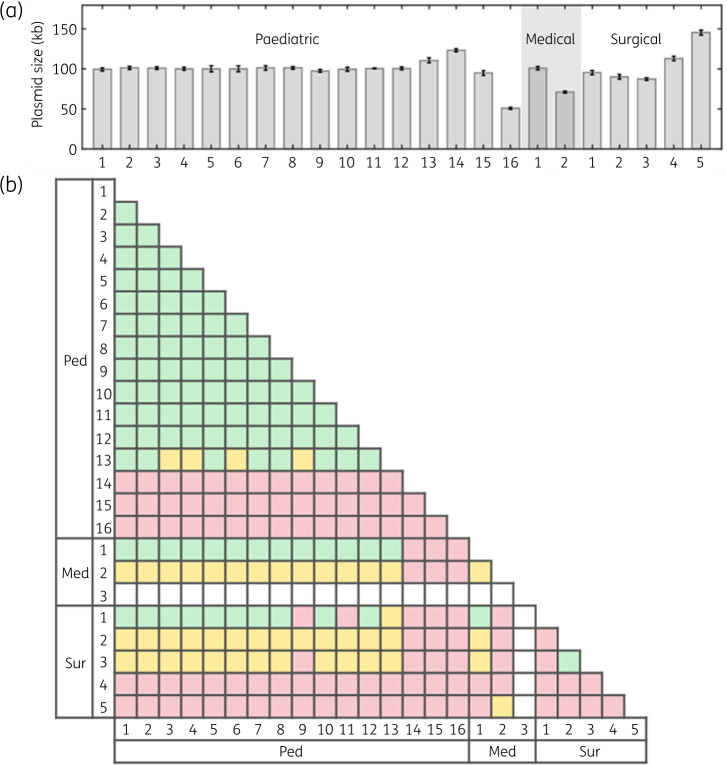
(a) Sizes of plasmids from the paediatric, medical and surgical wards. Plasmid sizes are estimated by comparing with spiked λ-DNA of known length. For each plasmid, at least six barcodes are collected and averaged for size estimation, and error bars represent one standard deviation. (b) Similarity matrix of plasmids. Plasmids are compared pairwise. If the barcodes are similar when stretched to the same size (*P*_comp_ < 0.01), they are considered complete matches and are labelled green. Barcode comparisons that do not match completely, but match when considering the size difference (*P*_part_ < 0.01), are considered partial matches and are labelled yellow. Red boxes represent *P *>* *0.01 for both comparisons.

The plasmids in all samples were analysed with ODM to obtain barcodes that could be used to compare plasmids between isolates. Figure 2(a) shows a histogram of sizes measured for the plasmid carrying the *bla*_CTX-M-15_ gene in all isolates. While the plasmids in many samples had a very similar size of around 100 kb, a few plasmids stood out with significantly different sizes, both larger and smaller, indicating that these plasmids were different from the common 100 kb plasmid.

To investigate the similarity between the plasmids, we compared the barcodes for the plasmids carrying *bla*_CTX-M-15_ in all isolates. The results are summarized in the matrix in Figure 2(b), where we place each pair of plasmids in one of three categories, and define *P *<* *0.01 as a significant match (see the Materials and methods section).^20^ The three categories were: matching when they were stretched to the same length (green in Figure 2b); matching but one was shorter than the other (yellow); or non-matching (red). Fourteen plasmid pairs were in the green category, suggesting they were identical, but there were also plasmids in the yellow and red categories. We discuss below these different groups of plasmids separately. In one of the samples (Med3) we did not find a plasmid encoding *bla*_CTX-M-15_, suggesting the gene was located on the chromosome (labelled white in Figure 2b).

**Figure 3.**
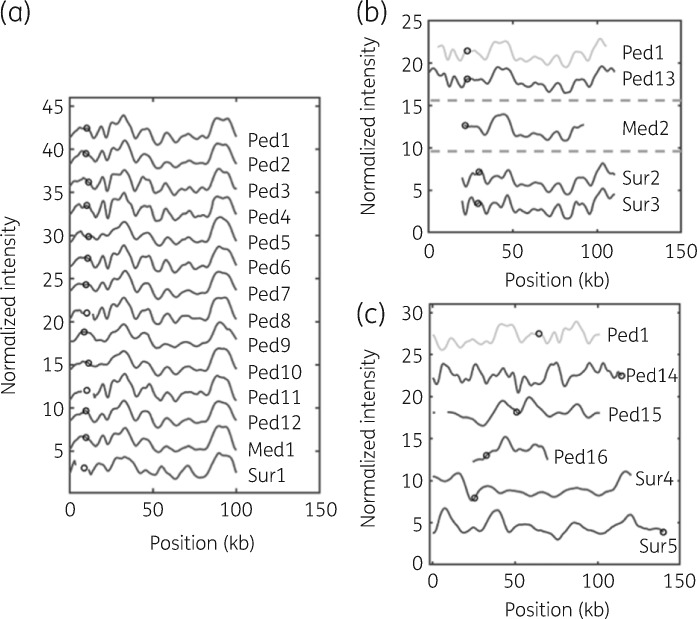
(a) Barcodes for all plasmids with a size of ∼100 kb with a *P* value below 0.01 when stretched to the same length. The barcodes are aligned to the best fit, and the location of the *bla*_CTX-M-15_ gene is marked with a circle on each barcode. The barcodes have been shifted vertically for clarity. (b) Barcodes for plasmids with different sizes that match partially to the shared barcode. Ped1 is plotted as a representative barcode of the ∼100 kb barcode (grey). Ped12 contains an insertion of ∼10 kb. Med2 contains an ∼30 kb deletion. Sur1, Sur2 and Sur3 contain deletions of ∼5, ∼10 and ∼10 kb, respectively. The barcodes are aligned to the best fit, and the location of the *bla*_CTX-M-15_ gene is marked with a circle on each barcode. The barcodes have been shifted vertically for clarity. (c) Barcodes for plasmids with different sizes that do not match the shared barcode. Ped1 is plotted as a representative barcode of the ∼100 kb barcode (grey). The barcodes are aligned to the best fit, and the location of the *bla*_CTX-M-15_ gene is marked with a circle on each barcode. The barcodes have been shifted vertically for clarity.

First, we compared the barcodes for all plasmids that had a size of around 100 kb (Figure 3a) and shown as green in Figure 2(b). Since we used Cas9 with a crRNA targeting *bla*_CTX-M-15_, we could directly pinpoint the presence of this gene on the plasmids. The barcodes were found to be highly similar, and the *bla*_CTX-M-15_ gene was located at the same position in all of them, clearly supporting that it was the same plasmid.

Ped13 was unique because it was significantly longer than the other plasmids (∼10 kb longer), but still obtained a *P* value below 0.01 when compared with many of the 100 kb plasmids in Figure 3(a). However, this *P* value dropped more than one order of magnitude when adjusting the lengths to their measured respective lengths. The *bla*_CTX-M-15_ gene was at the same location, further supporting that Ped13 featured the same plasmid, but with a ∼10 kb insertion. In Figure 3(b) the Ped13 plasmid is compared with the plasmid in Ped1 when stretched to their respective sizes.

Next, we investigated the plasmids that were labelled yellow in Figure 2(b) (Sur1, Sur2, Sur3 and Med2, Figure 3b). These were significantly smaller than the plasmid in Figure 3(a). The yellow plasmids were compared with the plasmid in Figure 3(a) by taking the respective lengths into account and using Ped1 as a representative barcode, allowing the shorter plasmid to be partially matching to the longer one.^20^ This analysis confirmed that the plasmids were indeed similar also in these samples, except that they all had deletions. With Ped1 as reference, Sur1 contained a deletion of ∼5 kb, Sur2 and Sur3 contained deletions of ∼10 kb and Med2 contained a deletion of ∼30 kb. These deletions also matched the sizes in the histogram in Figure 2(a). Importantly, these results confirmed that the barcodes from plasmids in Sur2 and Sur3 were identical. For Sur1 and Med2, the location of the *bla*_CTX-M-15_ gene along the barcode was the same as for the plasmids in Figure 3(a), but for Sur2 and Sur3 it was shifted ∼7.5 kb.

Figure 3(c) compares the barcodes for the plasmids carrying the *bla*_CTX-M-15_ gene in the five samples labelled red in Figure 2(b), with the barcode of Ped1 representing the plasmid in Figure 3(a). In all these samples, it is evident from the barcodes that the plasmids were not the same, in agreement with the statistical analysis. This was further emphasized by the fact that the *bla*_CTX-M-15_ gene location for the best matches in Figure 3(c) were also not correlated.

**Figure 4.**
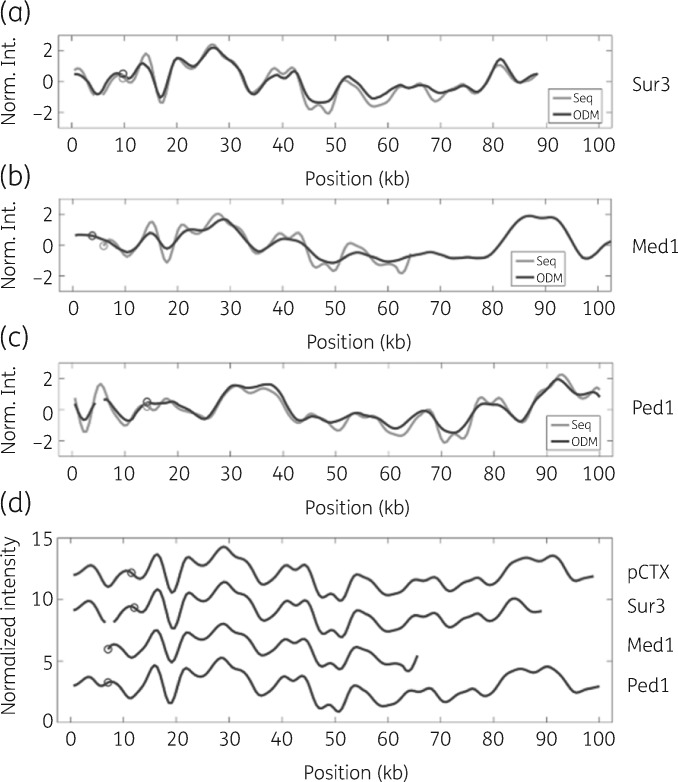
(a–c) Comparison between experimental barcodes (grey) and barcodes generated from nanopore sequencing data (black) for Ped1, Med1 and Sur3. (d) Comparison between the theoretical barcode for pCTX-M-15_22372 and the experimental barcodes for Ped1, Med1 and Sur3. The barcodes are aligned to the best fit, and the location of the *bla*_CTX-M-15_ gene is marked with a circle on each barcode. Norm. Int., normalized intensity.

As a control of our observations, we performed long-read nanopore sequencing of three of the isolates: Ped1, Sur3 and Med1 (Figure 4). For Ped1 and Sur3 the long-read sequencing yielded contigs covering the intact plasmids, and when transformed into theoretical barcodes they showed an excellent overlap with the corresponding ODM barcodes. For Med1 the full plasmid could not be assembled from the long-read sequencing, but a 59 kb contig containing the *bla*_CTX-M-15_ gene was identified. The theoretical barcode from this contig overlaps perfectly with a region of the plasmid in Med1. For all three plasmids the gene location matched perfectly between the theoretical and experimental barcodes.

The *bla*_CTX-M-15_-containing contigs obtained for the plasmids in Ped1, Sur3 and Med1 were compared with the RefSeq database (https://www.ncbi.nlm.nih.gov/refseq/). All three plasmids show great similarity (∼99.9%) to plasmid pCTX-M-15_22372 isolated in India. The theoretical barcode for pCTX-M-15_22372 is compared with the experimental barcodes for Ped1, Sur3 and Med1 in Figure 4(d), again showing great similarity. pCTX-M-15_22372 had the *bla*_CTX-M-15_ gene at the same position as Sur3, but shifted ∼7.5 kb compared with Ped1 and Med1. The observation that all plasmid sequences were so similar to pCTX-M-15_22372, and hence each other, answered the question remaining from the barcoding experiment—whether the Sur2 and Sur3 plasmids were related to the main outbreak plasmid. The barcodes indicated this, but the fact that the resistance gene was shifted 7.5 kb did not agree with this observation. From the long-read sequencing data, we could confirm that the plasmids were highly similar, suggesting that they are of the same origin.

**Figure 5.**
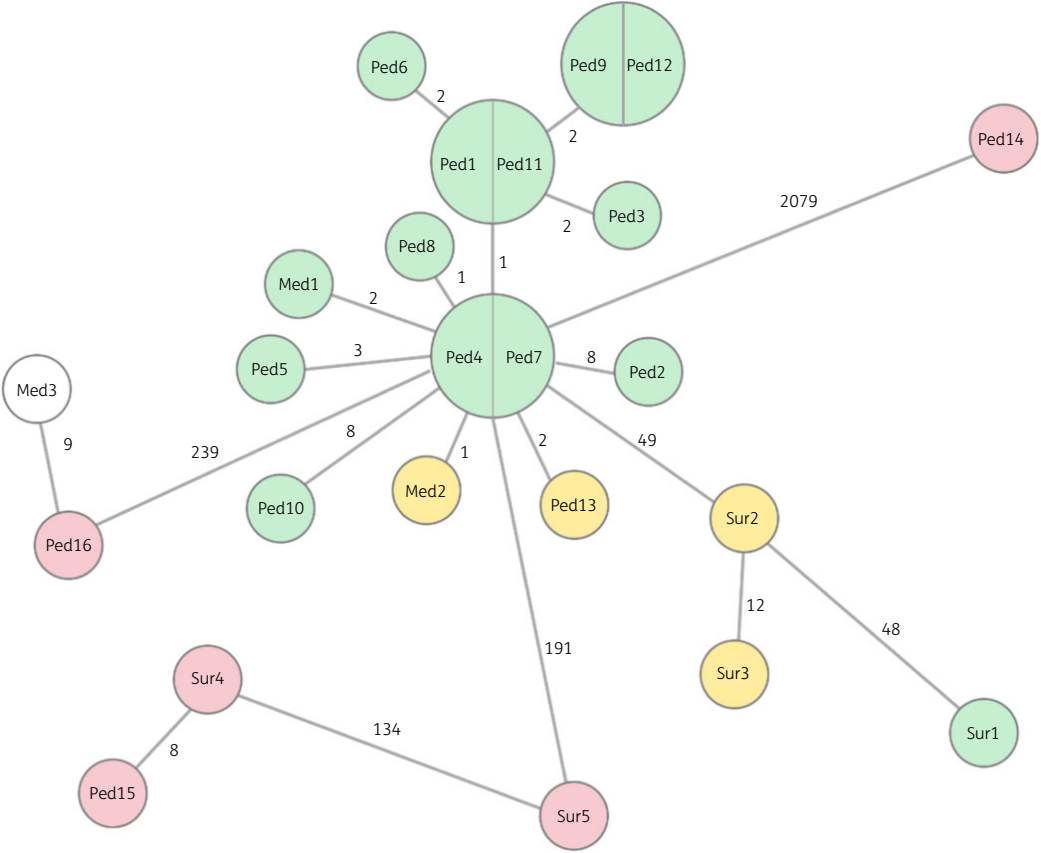
Minimum spanning tree (GrapeTree) of all strains based on cgMLST. The numbers on the solid lines correspond to the number of alleles that differ between each strain pair. The colours represent the same as in Figure 2.

The plasmid barcoding data showed that the same plasmid was found in 18 different patients in three different wards, suggesting that the same bacterial strain had spread between the three wards. We returned to the NGS data and conducted an in-depth analysis of the chromosomal DNA. This analysis is summarized in the core genome (cg)MLST-based minimum spanning tree in Figure 5, where it is correlated to the plasmid typing. The plasmid typing agreed very well with the cgMLST data; the samples with similar plasmids also have a core genome with a similarity of at least 99.5%. All these strains shared similar resistance profiles [*bla*_CTX-M-15_, *bla*_OXA-1_, *bla*_CMY-2_, *aac(6′)-Ib-cr*, *aad5*, *catB3*, *su1/sul2*] as extracted from ResFinder, and none of them encoded any of the virulence genes investigated. Samples that showed no similarity in the plasmids all showed a significant difference also in the cgMLST.

## Discussion

We have in several previous studies reported the use of ODM to characterize and trace bacterial plasmids collected in hospital settings.^20^^,^^24^ Our recent demonstration that Cas9 can be used to identify specific genes further improves the method and enables analysis of plasmids in great detail in a single experiment.^25^^,^^27^ The study of plasmids from isolates collected in a rural hospital in Ethiopia using ODM presented here highlights the usefulness of the technique in different ways.

First, the ODM analysis identified a large group of isolates that contained a plasmid of the same size, where the *bla*_CTX-M-15_ gene was located at the same position along the plasmid. We would like to stress the advantage of obtaining the barcodes rather than only the plasmid sizes.^30^ The barcodes can both differentiate plasmids when plasmid sizes are similar and also identify the same plasmids with different sizes when an insertion or deletion occurs, which is common in plasmids. The exact location of such structural variations can, as described in a previous study, be confirmed with PCR.^27^ Large-scale variations (5–30 kb identified here), which are challenging to short-read sequencing techniques, are rapidly detected with ODM. This, in turn, means that transmission routes where such structural changes have occurred can also be identified. The results were confirmed with long-read sequencing of a small fraction of samples. This might become a general strategy, where representative samples are identified with ODM and analysed in further detail with long-read sequencing.

Second, we wish to highlight the possibility of using plasmid typing as a proxy for identifying clonal spread of bacteria and, in turn, ongoing outbreaks. While plasmids can also spread horizontally, the identification of the same plasmid in as many as 18 different patients in a hospital is a reliable indicator of an ongoing outbreak, as plasmid transmission will also only happen whenever strain transmission occurs. Plasmid typing might become a straightforward method to detect such outbreaks, especially when new methods for typing of plasmids, such as the one presented here, are being developed. Another possible explanation for the high prevalence of a bacterial strain with an identical plasmid in the hospital setting could be that this particular strain is widespread in the community. We want to stress that this could not be resolved with the ODM technique, but it would also be highly challenging with competing techniques like NGS. In this particular study there was a strong overlap between similarity of the plasmids and the cgMLST data, where a clone of *E. coli* ST410 was shown to have the same plasmid. Compared with cgMLST, we foresee that plasmid typing with ODM might, at least in some settings, be a more accessible and faster technique that does not require NGS or bioinformatics analysis. Plasmid ODM also directly confirms that the same plasmid is present in all strains from an outbreak.

Third, we would like to stress the potential future use of the method in LMICs. The barcodes generated using the principle presented here rely on variations in a strong emission signal, which is in stark contrast to most competing techniques, where specific sequence motifs are labelled with one or a few fluorophores.^21^ While not yet implemented, we believe that the presented assay can be transferred to simple and cheap microscopy formats. YOYO-stained molecules have been visualized using a fluorescence microscope based on a smartphone camera, which will be an appealing format for LMICs.^31^

### Conclusions

This study analysed plasmids from isolates collected from three different wards in a rural Ethiopian hospital using both ODM and NGS. The results demonstrate a potentially ongoing outbreak of *E. coli* ST410 where the same plasmid was found in three different wards. Small changes in the plasmid due to insertions and deletions were found in some of the plasmids. The NGS data correlate well with the ODM data, where strains with the same plasmid are closely related while strains that were less related also did not harbour the same plasmid. We propose that ODM of plasmids can be a useful tool to detect transmission of bacteria and plasmids in hospital settings and that the method might be applicable in LMICs.
